# The complete mitochondrial genome of the critically endangered diving beetle *Dytiscus sharpi* (Coleoptera: Dytiscidae)

**DOI:** 10.1080/23802359.2019.1630333

**Published:** 2019-07-12

**Authors:** Nobuaki Nagata

**Affiliations:** Division of Collections Conservation, National Museum of Nature and Science, Ibaraki, Japan

**Keywords:** Complete mitogenome, diving beetle, Dytiscinae, *Dytiscus sharpi*

## Abstract

The diving beetles *Dytiscus sharpi* is one of the most critically endangered species in Japan, caused by excessive capture and destruction of their habitats. Here, the complete mitochondrial genome sequence (mitogenome) of *D. sharpi* is described. The entire mitogenome sequence was 19,476 bp long, containing 13 protein-coding genes, 2 ribosomal RNA genes, 22 transfer RNA genes, and 1 control region. The average A + T content of mitogenome was 81.3%. This is the first description of a complete mitochondrial genome of *Dytiscus*, and the second of Dytiscinae. The primary genetic data obtained in this study is expected to contribute to conservation genetic studies using various genetic analysis methods, including environmental DNA.

*Dytiscus sharpi* is one of the largest diving beetles distributed in the Japanese archipelago and inhabits muddy wetlands. In Japan, diving beetles, a member of the Dytiscinae, are declining rapidly and 14 species are listed on the Japanese Red List as endangered species. *Dytiscus sharpi* has been rated IA and has been designated as a target species under the species protection law in Japan, ‘Act on Conservation of Endangered Species of Wild Fauna and Flora’, from 2011. In recent years, several studies of genetic relationships among regions (Nagata et al. 2018) and ecology (e.g. Inoda 2011) have been reported. However, as a small number of mitogenomes of diving beetles have been characterized, phylogenetic analysis based on mitogenome and design markers for environmental DNA analysis of diving beetles containing *D. sharpi* are difficult.

In this study, the complete mitochondrial genome (mitogenome) of *Dytiscus sharpi* is characterized using DNA from a previous study (No. 6 from population F in Niigata prefecture (Nagata et al. 2018)). The specification of detailed sampling sites is not permitted for conservation purposes. Total DNA was stored in National Museum of Nature and Science (NSMT-DNA41512). Mitogenome was amplified in two fragments by PCR except DNA barcoding region. Then, two fragments were sequenced by pair-end sequencing using Miseq sequencer (Illumina, San Diego, CA). The total reads were assembled using CLC Genomic work bench (Qiagen, Hilden, Germany). The control region was detected using the primer walking method applying the Sanger sequence using BigDye 3.1 and an ABI 3500 sequencer (Thermo Fisher Scientific, Waltham, MA). Circularity was checked visually, and the mitogenome was annotated using MITOS WebServer (Bernt et al. 2013)

The size of entire mitogenome sequence of *Dytiscus sharpi* was found to be 19,476 bp long (GenBank/DDBJ/EMBL Accession number LC466129), containing 13 protein-coding genes (PCGs), 2 ribosomal RNA (rRNA), 22 transfer RNA, and 1 control region (CR). The overall base composition was extremely AT-rich, with 43.0% for A, 38.3% for T, 7.8% for G, and 10.9% for C.

The phylogenetic trees of Dytiscidae based on 13 PCGs and 2 rRNA’s were reconstructed by maximum likelihood and neighbor-joining methods using RAxML (Stamatakis 2015) and PAUP* 4.0 beta (Sofford 2002), respectively. In these analyses, the following eight diving beetles and one carabid ground beetle were included as an outgroup, *Acilius* sp, *Colymbetes* sp., *Hydroporus* sp., *Liopterus* sp. (Linard et al. (2016)), *Agabus uliginosus*, *Hygrotus nigrolineatus* (Hunter et al. (unpublished)), *Limbodessus palmulaoides*, *Paroster macrosturtensis* (Hyde et al. (2017)), and *Carabus lafossei* (Liu et al. (2018)). Both phylogenetic trees are similar to each other (Figure 1), and Dytiscinae, including *Dytiscus sharpi*, were monophyletic. The genetic distance within Dytiscinae was 13.49% at the COI and 9.84% at the 16S rRNA.

**Figure 1:**
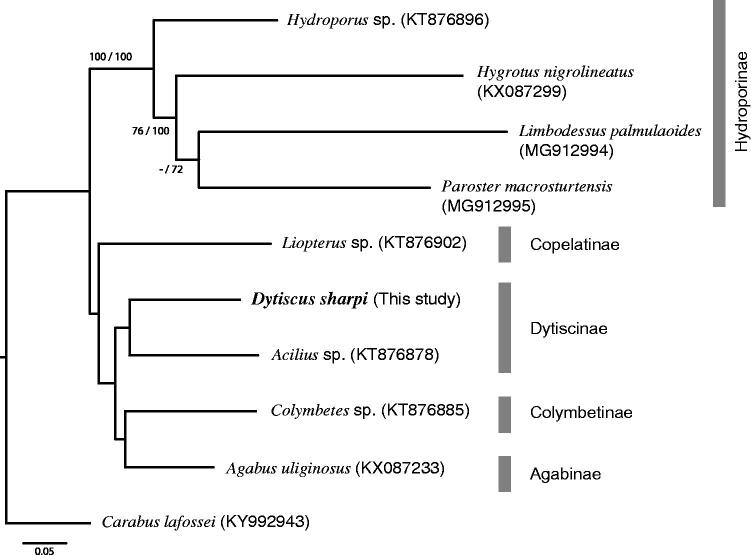
The maximum likelihood phylogenetic tree of Dytiscidae based on 13 protein coding genes and two rRNA. The number beside each node represent bootstrap values in percentage based on 1000 replication (maximum likelihood estimation followed by neighbor-joining estimation). The number after the species name is the GenBank Accession number.

This is the first description of a complete mitochondrial genome of *Dytiscus*, and the second for within subfamily Dytiscinae. The mitogenome of *D. sharpi* obtained in this study is expected to contribute not only to conservation genetic studies using various genetic analysis methods, including eDNA but also for the clarification the phylogeny of Dytiscidae.
